# The increasing role of tree disease and decreasing influence of anthropogenic management over 50 years of woodland dynamics

**DOI:** 10.1098/rspb.2025.0554

**Published:** 2025-06-11

**Authors:** Fiona Seaton, Claire Wood, Karen Hornigold, Keith Kirby, Chris Nichols, David Jam, Emma Dear, Adam Kimberley, Simon Smart

**Affiliations:** ^1^ UK Centre for Ecology & Hydrology, Lancaster, UK; ^2^ Woodland Trust, Grantham, UK; ^3^ University of Oxford Department of Biology, Oxford, UK; ^4^ Forestry Commission, Ludlow, UK; ^5^ Natural England, Peterborough, UK

**Keywords:** ash dieback, *Hymenoscyphus fraxineus*, forest, species richness, tree regeneration, vegetation, deer grazing, forb cover, grass cover, *Rubus fruticosus*

## Abstract

Woodlands are under threat from a variety of global change stressors, and understanding the main effects and interactions between these is critical for their protection. Here, we analyse vegetation change over 50 years within approximately 100 broadleaved woodland sites across Great Britain from 1971 to 2022 and quantify the interactions between management history, deer herbivory and ash dieback. We find an overall trajectory towards a less diverse, more shade-adapted ground flora which has recently been locally disrupted by ash dieback. Plots with evidence of ash dieback have higher forb cover and ground flora richness relative to plots without dieback. However, this effect of ash dieback was shown mainly where there was high grazing risk; the grazing reduces the vigour of generalist understorey shrubs which can also lead to lower tree regeneration. Our results reveal how woodland dynamics have been shaped initially by a response to a reduction in interventionist management and then by disturbance driven by high herbivory risk and a novel tree disease.

## 1. Introduction

Woodlands harbour biodiversity, act as cultural touchstones, provide timber, regulate water flows and influence climate [1]. Woodland restoration and management have been identified as key components of adapting to and mitigating the effects of the climate crisis [2]. Conservation of long-established broadleaved woodlands is of particular importance as these support many rare species, have disproportionate impact on biogeochemical cycles, have greater cultural significance than recently created woodlands, and act as reservoirs of species that may then colonize restored or newly created woodlands [3]. Within the past hundred years, there has been a change in how existing woodlands are managed across Europe, with a loss of coppice and a shift towards high forest within remaining woodland fragments [4–6]. In addition, woodlands within Great Britain were affected at a national scale by a mass felling of trees during and just after World War II. Subsequently, in many woods, gaps were filled by the planting of fast-growing non-native coniferous species; where woods remained predominantly broadleaved, the gaps were filled by natural regeneration, generally with little other active management because of the poor economic returns from broadleaved forests and increasing urbanization [7,8]. The decline in active management, particularly of coppicing, led to a general increase in shading across woodlands and a loss of permanent open spaces. This led to a decrease in richness of vascular plant species, including declines in many woodland specialist species [3]. Patterns of tree regeneration shifted, as increased shading reduced the regeneration of shade-intolerant species leading to long-term shifts in canopy composition [4,9]. This change in management coincided with increasing pressures on woodland ecosystems from other sources, such as pollution, climate change, land use change, poor ecological connectivity and the spread of novel tree diseases [3,10].

A major driver of change in European woodlands in the last 30 years has been ash dieback (caused by the fungus *Hymenoscyphus fraxineus*), which after being first observed in Eastern Europe in the 1990s has spread westwards across the continent, affecting woodland ecosystems [11]. Ash dieback was first confirmed in Great Britain in 2012, but there is evidence that it had been present since 2004 and potentially imported in the 1990s [12]. Some initial estimates were that ash trees would be wiped from the European landscape, but more recent reports indicate some resilience within ash communities [13,14]. Currently, the long-term impacts upon ash are unclear and may depend upon other factors such as soil conditions and deer browsing [13–17]. Loss of ash has been predicted to result in declines in woodland biodiversity and changes in nutrient cycling due to the unique characteristics of ash within European woodlands and the high numbers of species that depend upon ash [11,18]. Compared to other native European deciduous species that may replace it, ash casts lighter shade, has more decomposable litter, has greater fine root biomass and hydraulic conductivity and has distinct soil nutrient and microbial community profiles [19–21]. Not only will the loss of ash itself have long-term impacts upon woodland ecosystems as it is replaced by other species, there will also be short- to medium-term impacts as defoliation and tree death open gaps in the canopy. Historically, as discussed above, woodlands had many canopy gaps due to active management which have since become less common. Therefore, the loss of ash trees could potentially mimic the impacts of historical management as woodlands shift away from high forest towards a more open structure.

Understanding the impacts of ash dieback upon woodland dynamics requires taking into account other major drivers of woodland vegetation dynamics, such as climate change and the dramatic expansion of deer populations [22,23]. Climate change is causing shifts in forest plant communities towards species adapted to warmer environments, although this process may be moderated where canopy closure leads to less warming of the microclimate at ground level [23,24]. The sudden creation of canopy gaps, whether from ash dieback or from extreme weather events [25], could increase the vulnerability of understorey plant communities to climate change. Deer grazing can lead to lower vegetation cover but also alter vegetation composition, preventing plants such as bramble (*Rubus fruticosus* agg.) from dominating and hence increasing ground flora richness [26–29]. Deer grazing also reduces tree regeneration, which could interact with the short-term changes caused by canopy opening from ash dieback resulting in differing trajectories in woodland composition and canopy structure.

Changes in woodland ecosystems in response to environmental stressors may occur over decades, while historical legacies may persist for hundreds to thousands of years [30,31]. Understanding how modern threats are affecting woodland ecosystems therefore needs long-term studies of woodland state and change. Here, we present data gathered from ~100 long-established woodlands across Great Britain from 1971 to 2022 and evaluate how woodland plant communities have changed over the 50 years. The ‘Bunce’ woodland survey was first carried out in 1971, targeting 103 sites representative of a wider sample of over 2000 broadleaved woodlands across Great Britain [32,33]. These sites were then revisited in both the early 2000s and 2020s and surveyed using the same methodologies to enable measurement of change over five decades. We quantify plant community change across the Bunce woodland sites each with up to 16 plots measured in 1971, 2000−2003 and 2020−2022, and the roles of ash dieback, deer grazing and climate in causing these changes.

Our hypotheses were that:

— The woodlands would have continued along a stand-development pathway with fewer, larger stems and a less diverse ground flora adapted to shadier conditions.
— Ash dieback, by canopy morbidity and reduced leafing, would partially offset these developmental changes with greater ground flora diversity within plots with ash dieback.
— The effect of ash dieback would be affected by deer grazing, as deer affect both tree regeneration and ground flora cover, particularly of highly palatable species such as bramble.

## 2. Material and methods

### (a) Field survey

The first ‘Bunce’ woodland survey in 1971 comprised 103 sites representative of a wider sample of 2453 broadleaved woodlands across Great Britain, in each of which sixteen 14.1 m × 14.1 m randomly placed quadrats were recorded. The 2001 re-survey visited all the sites, but 38 plot positions were no longer wooded and so not recorded. For this resurvey, 16 sites were surveyed in 2020, 39 sites in 2021 and 42 sites in 2022. In total, 97 of the original 103 sites were resurveyed. The original 16 plots were relocated and resurveyed, where possible, in each site using the same methods as the previous surveys [33,34]. Except where permission was refused or the site was destroyed, a 14.1 m by 14.1 m (200 m^2^) plot was established as close to the original plot location as possible, see Kirby *et al.* [32] for a quantification of the plot relocation error from the original survey and Smart *et al.* [34] for a description of the methods used to ensure relocation accuracy within the most recent survey. All plants within the ground flora were recorded, and an estimate given of their cover (to the nearest 5%). Trees within the plot were also recorded, with diameter at breast height (DBH) estimated within 5 cm bands. Other plot attributes recorded by the surveyor included the presence of ash dieback and evidence of historic and recent canopy gap creation through felling and coppicing. Ownership of the sites was assigned as either private (an individual, farm or estate), forestry (an enterprise whose primary focus was timber production) or conservation (nature reserves, councils, statutory agencies and trusts motivated at least partly by conservation objectives).

### (b) Calculation of response variables

Summary metrics for the ground flora of each plot were calculated including: species richness, Shannon diversity, ancient woodland indicator (AWI) richness, forb cover (defined as non-woody flowering plants), fern cover, grass cover (defined as all Poacaea), woody plant cover and bramble (*Rubus fruticosus* agg.) cover (since the latter is a widely distributed understorey dominant in many British woodlands). Species richness was calculated at both the plot level and the site level. In addition, three summary metrics based upon the tree data were calculated: tree richness, stem count and mean basal area. Evidence of tree regeneration was also recorded as a binary variable by the surveyors; we include the presence of regeneration of any tree species (total regeneration) and regeneration of ash trees. Regenerating stems were defined as >0.25 and ≤1.3 m. Shannon diversity was calculated using the proportion of vascular plant cover. AWI lists were compiled from various regional sources and the appropriate regional list used per site [35–38]. All summary metrics were limited to vascular plants only due to inconsistencies in the recording of bryophytes between survey years. Some species were also aggregated into amalgamated groupings due to the differences in levels of taxonomic identification between years (e.g. combining *Viola riviniana* and *Viola reichenbachiana*).

### (c) External data

The average summer rainfall, winter rainfall, winter average minimum temperature and summer average maximum temperature were calculated over the 20 years pre-survey for all plots based on the 1 km HadUK-Grid measurements for the Met Office [39]. Deer risk at the site level was categorized into low, moderate and high for all sites based upon a combination of expert assessment for sites in Wales, the indicative Deer risk data layer for England available from the Forestry Commission [40], and by applying the modelled deer impact algorithm in Spake *et al.* [41] for sites in Scotland.

### (d) Statistical modelling

The statistical analyses can be categorized into three groups: (i) change over time; (ii) ash dieback and deer effects; and (iii) multivariate analyses. The first two of these categories are both univariate, i.e. one model per response metric, and are focused on (i) establishing whether the response metrics change over the three surveys and (ii) whether any changes over time were affected by ash dieback and deer grazing. No model selection was performed for these categories, and an estimated marginal means approach was used to estimate changes between years and treatments. Analyses within the third category consisted of multivariate models, which each had multiple response variables selected due to their importance in woodland dynamics. This allowed us to evaluate the interactions between vegetation metrics to establish if the impacts of ash dieback and deer grazing acted directly upon ground flora richness and tree regeneration or were mediated by changes in other woodland properties. Multiple model configurations were fit and then compared using cross validation to establish if all links between the different variables were needed. All statistical modelling was carried out within a Bayesian hierarchical framework, using the brms R package as an interface to Stan [42,43]. The analysis was carried out within R v. 4.4.0, brms v. 2.21.0 and rstan v. 2.36.6.

#### (i) Change over time

The summary metric of interest was modelled as a function of survey year (treated as categorical), with the day of year the plot was surveyed also included as a predictor, and a hierarchical effect of plot nested within site on the model intercept. For the change over time models only (i.e. not the models with explanatory variables included), the effect of the survey year was also allowed to vary by site in a hierarchical effect.

Count variables were modelled using the negative binomial family with the exception of tree richness which was modelled using the Poisson family as there was no improvement in model fit when using the negative binomial family, and AWI richness which was modelled as a binomial variable with number of trials equal to total richness within the plot. Model fit was assessed using graphical posterior predictive checks, and all count variables (other than AWI richness) were modelled using both a Poisson and a negative binomial family and the relative fits of the model to the data were compared graphically.

Shannon diversity was modelled using the Gaussian family, regeneration variables using the Bernoulli family, and cover variables using a hurdle gamma family. The hurdle gamma family was chosen for cover as the total cover of any growth form was bounded at zero, but could go above 100% due to the different canopy levels included in the calculation. Within the hurdle gamma models, the mean and hurdle parts of the model were modelled using the same predictor variables.

Weakly informative priors were used for all model parameters, these were tested by sampling only from the prior and examining the prior predictive distribution. The priors used for each model are detailed in electronic supplementary material, table S1. Model convergence was assessed by having Rhat less than 1.01 and both bulk and tail effective sample sizes greater than 1000. Fit to the data was assessed using graphical posterior predictive checks and the model residuals were checked for anomalous results by site and region. Comparisons between the years were conducted using estimated marginal means, and the 95% HPD of each comparison was calculated [44]. The tidybayes package was used to plot the 50%, 80% and 95% intervals of each effect [45].

#### (ii) Ash dieback and deer effects

For sites that had ash present in the DBH data in the most recent survey (86 sites), we categorized plots into no ash present (669 plots), ash present but no dieback (328 plots) and ash dieback present (311 plots). For site-level ground flora richness only, the ash dieback effect was modelled as a function of the proportion of plots with ash dieback. The effect of ash dieback was modelled as an interaction effect on year. Deer impact was modelled as an ordinal effect (low < moderate < high) that interacted with year [46]. Whether the effects of ash dieback varied by deer risk was tested using an interaction model, with ash dieback and deer risk being modelled as above.

#### (iii) Multivariate model

To investigate the way in which the different components of the understorey community interact in their response to ash dieback, deer grazing and climate a multivariate model (i.e. with multiple response variables) was fit using brms. Within this model bramble cover, forb cover, grass cover, total tree regeneration and ground flora species richness were all modelled together as a function of survey year, ash dieback (affecting forbs, tree regeneration and species richness only), deer risk (affecting bramble, grass cover, tree regeneration and species richness only), day of year, climatic variables and a site/plot hierarchical effect on the intercept. No ash dieback effect on bramble or deer effect on forbs were included due to no evidence for these metrics responding to these drivers in the univariate models. Forb and grass cover were also modelled as a function of bramble cover, and species richness and tree regeneration as a function of bramble, grass and forb cover. Response families and number of iterations were as within the individual models, priors are detailed in electronic supplementary material, table S2. As the responses were non-Gaussian, no residual correlation between the responses could be included as a parameter within the model using brms.

To test our hypotheses, we fit multiple multivariate models with differing configurations, which were then compared for their predictive performance using 10-fold cross validation, with site being used as a grouping variable. As there were strong correlations between the four climate variables included here (winter rainfall, summer rainfall, summer maximum temperature and winter minimum temperature), different combinations were included to see which showed the best predictive performance. These combinations were: all four climate variables; winter rainfall and winter minimum temperature; summer rainfall and summer maximum temperature; and no climate variables. Once the best combination of climate variables by predictive performance was identified, then the performance of the model without direct links from deer or ash dieback to species richness or tree regeneration was assessed. To assess whether ash dieback impacted species richness rather than just richness differing between ash plots versus non-ash plots, the ash woodland versus no ash woodland distinction was kept as a predictor of species richness, but the dieback versus no dieback was removed.

## 3. Results

### (a) Change over time

**Figure 1.**
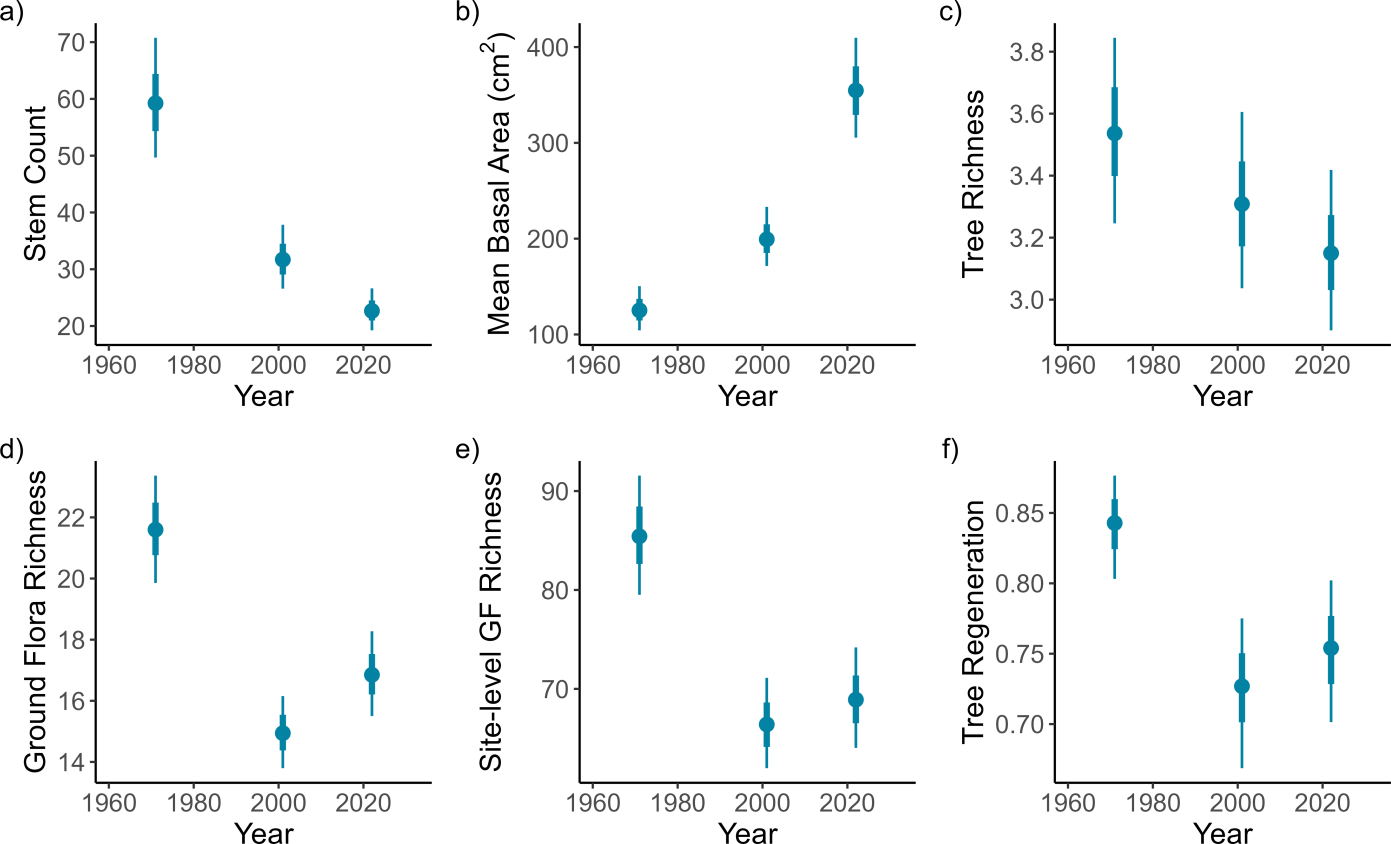
Changes in the number of tree stems (a), mean basal area of those stems (b), tree richness (c), ground flora richness (d), site-level ground flora richness (e) and tree regeneration (f) from 1971 to 2001 to 2022. Metrics are calculated at the plot level unless otherwise stated. The dot and the thick and thin vertical lines represent the median, 66% interval and 95% interval respectively of the model estimate.

From 1971 to 2022 there has been a shift to fewer, larger trees across the surveyed woodlands: in 2022, there were only 40% of the number of stems in 1971, but those stems were about 2.8 times as large in basal area (figure 1; electronic supplementary material, table S3). During this period, there was a decrease in interventionist management across the sites: while there was evidence of historic coppicing and/or tree clearing in around 60% of the plots, only 10% of plots showed evidence of recent canopy gap creation in the 2022 survey compared to 18% of plots in the earlier surveys. Woodlands that were owned privately or by conservation charities both showed a halving in the number of plots that had evidence of recent canopy gap creation from 1971 to 2022, from ~9% to ~5% of plots, whereas plots owned by forestry-focused enterprises had a relatively constant rate of gap creation of around 25% between 1971 and 2022. Deadwood increased in all ownership categories from 1971 to 2022, rising from 60% to 90% of plots having some type of deadwood present. Tree regeneration initially declined from 1971 to 2001 then increased slightly, however ash regeneration declined slightly from 1971 to 2022 (figure 1; electronic supplementary material, table S3).

Concurrent with these changes in canopy structure there have been changes in both tree and ground flora richness. Tree species richness has decreased by around 10% from 1971 to 2022, while ground flora richness declined by around 30% from 1971 to 2001, then increased by around 10% from 2001 to 2022 resulting in an overall decline of around 20% (figure 1; electronic supplementary material, table S3). The same trend was shown by Shannon diversity (electronic supplementary material, table S3). The increase in ground flora richness at the plot level from 2001 to 2022 was not matched by an increase in ground flora richness at the site level which remained stable at around 65−70 per site relative to the total 795 vascular plant taxa across the entire survey (figure 1; electronic supplementary material, table S3).

There was also no change in AWI proportions at either the plot or site level (electronic supplementary material, table S3). The impact of increased shading upon the ground flora can be seen through the increasing cover of ferns (up by ~2.5 percentage points) which are more shade tolerant than the other plant groups such as grasses, forbs and woody plants which showed no change (electronic supplementary material, figure S1 and table S3). The date on which the plot was surveyed affected all of the ground flora variables other than AWI proportion, woody plant and bramble cover, with species richness, Shannon diversity and forb cover decreasing later in the season, and fern and grass cover increasing.

### (b) Ash dieback effects

**Figure 2.**
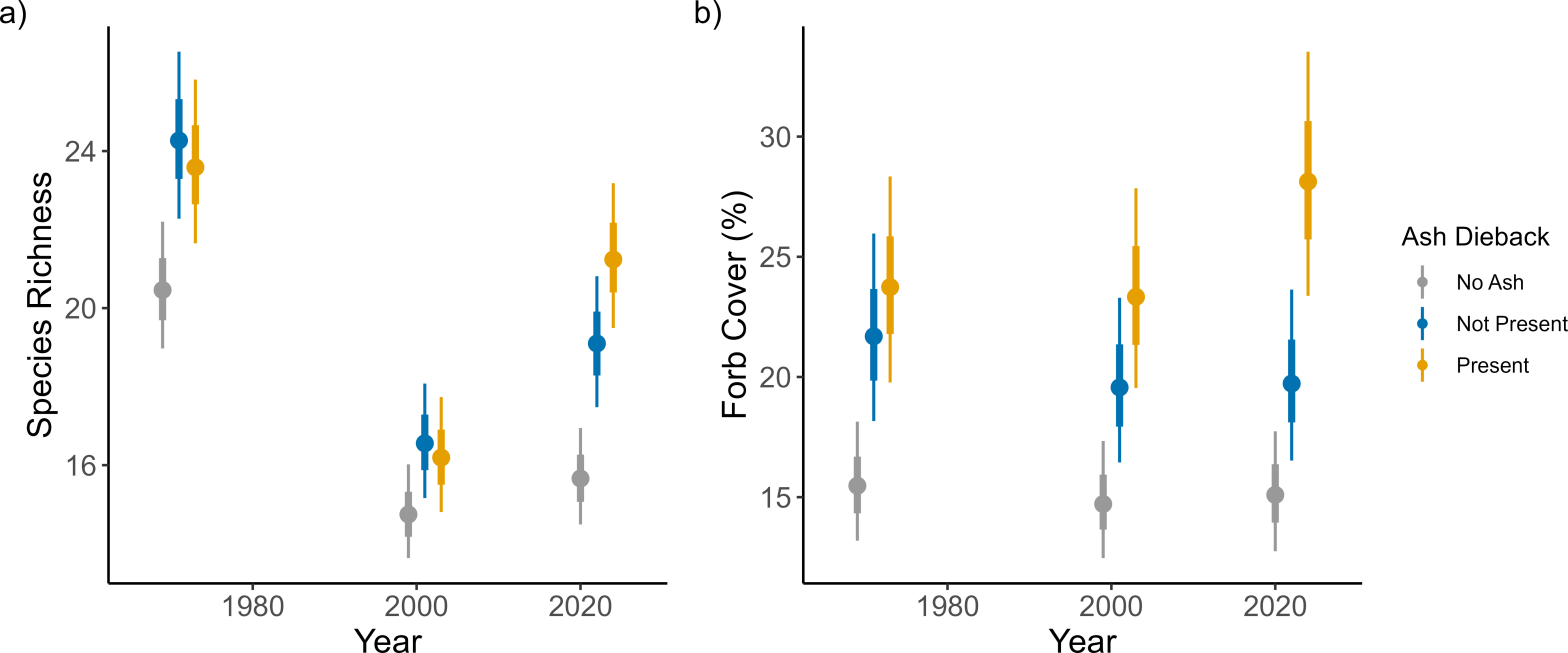
The different changes over time in ground flora richness (a) and forb cover (b) within plots that have ash dieback (yellow), plots that have ash present but no ash dieback (blue) and plots that have no ash present (grey). The dot and the thick and thin vertical lines represent the median, 66% interval and 95% interval respectively of the model estimate.

Plots that had ash dieback had higher ground flora richness in 2022 than plots without ash dieback, yet these plots did not differ in ground flora richness in the surveys prior to the arrival of the disease (figure 2a; electronic supplementary material, table S4). Plots without ash (but within sites that did have ash somewhere) had lower ground flora richness overall and did not change from 2001 to 2022. Plots with ash present but no dieback increased in ground flora richness over that time period, but not as much as plots with ash dieback present. Plots with ash dieback present had the greatest increase in Shannon diversity from 2001 to 2022, however, there was still no clear difference between plots with and without ash dieback in the 2022 survey (electronic supplementary materials, figure S2a and table S4). This indicates that the increase in ground flora richness is driven by an increase in the number of subordinate species by plot. The proportion of plots per site with ash dieback also had no effect upon the site-level ground flora richness within any year, indicating that there was no change in the overall species pool per site caused by ash dieback. The proportion of AWIs increased in plots with ash dieback, however this occurred from 1971 to 2001 and resulted in no overall difference in AWI proportion with or without ash dieback in the most recent two surveys (electronic supplementary materials, figure S2b and table S4). Forb cover increased under ash dieback, with ash plots without ash dieback having on average 8 percentage points less forb cover than in plots with ash dieback (figure 2b; electronic supplementary material, table S4). The cover of ferns, grasses, woody plants and bramble showed no effect of ash dieback (electronic supplementary material, table S4).

### (c) Moderating effect of deer grazing on ash dieback

**Figure 3.**
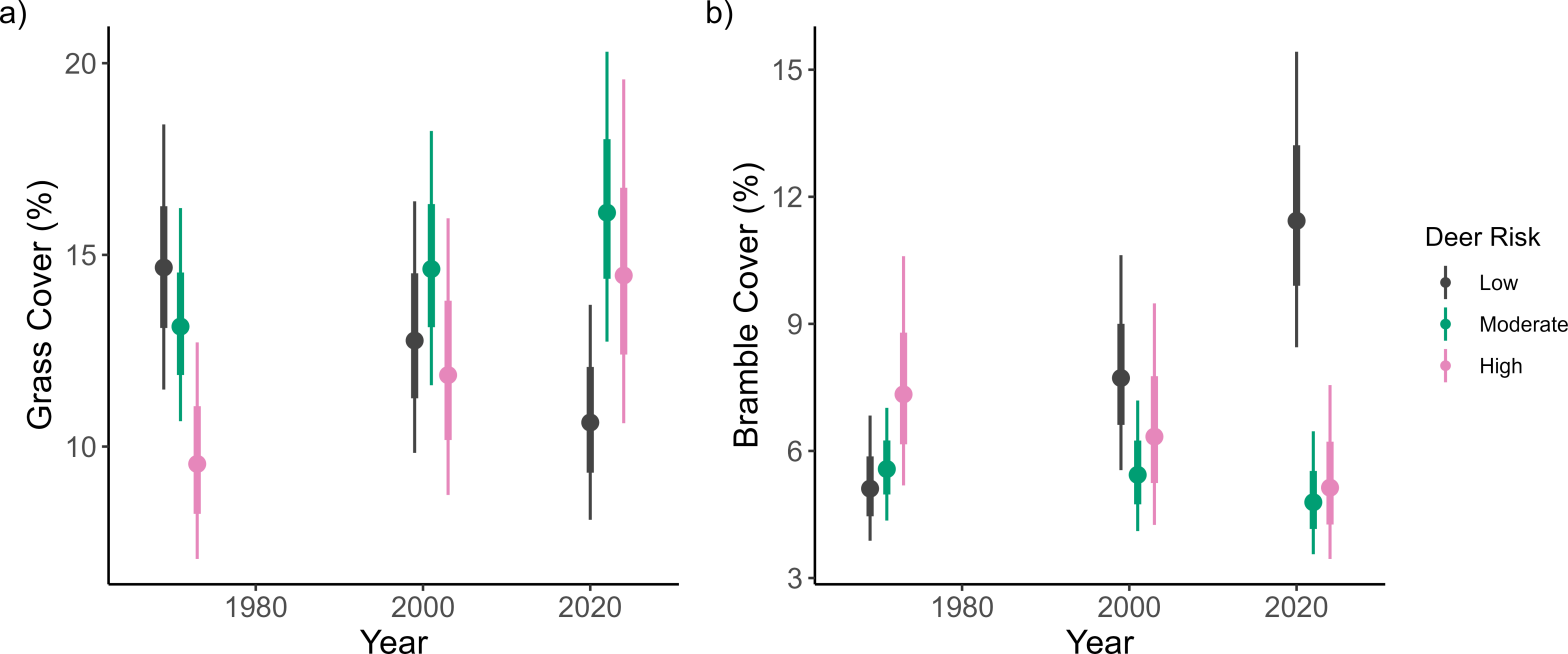
Impact of deer risk on plot-level grass cover (a) and bramble cover (b). The dot and the thick and thin vertical lines represent the median, 66% interval and 95% interval respectively of the model estimate.

Over the entire survey, there were 36 low, 35 moderate and 32 high risk for deer grazing sites. Deer grazing risk had a strong influence on understory components, with a doubling in bramble cover and a halving of grass cover in low deer risk sites from 1971 to 2022, no overall change in moderate deer risk sites and a slight decrease in bramble and doubling in grass cover in high deer risk sites (figure 3; electronic supplementary material, table S5). Deer risk was also associated with changes in tree DBH and tree regeneration. Trees were on average larger in high deer risk sites, although there was no association with stem count (electronic supplementary material, table S5). High deer risk sites had higher total tree regeneration in 1971 and then had a greater decline up until 2022 which removed this differential (electronic supplementary material, table S5). Ash regeneration was on average higher in low deer risk sites in the two most recent surveys, but confidence in this estimate is low (electronic supplementary material, table S5).

**Figure 4.**
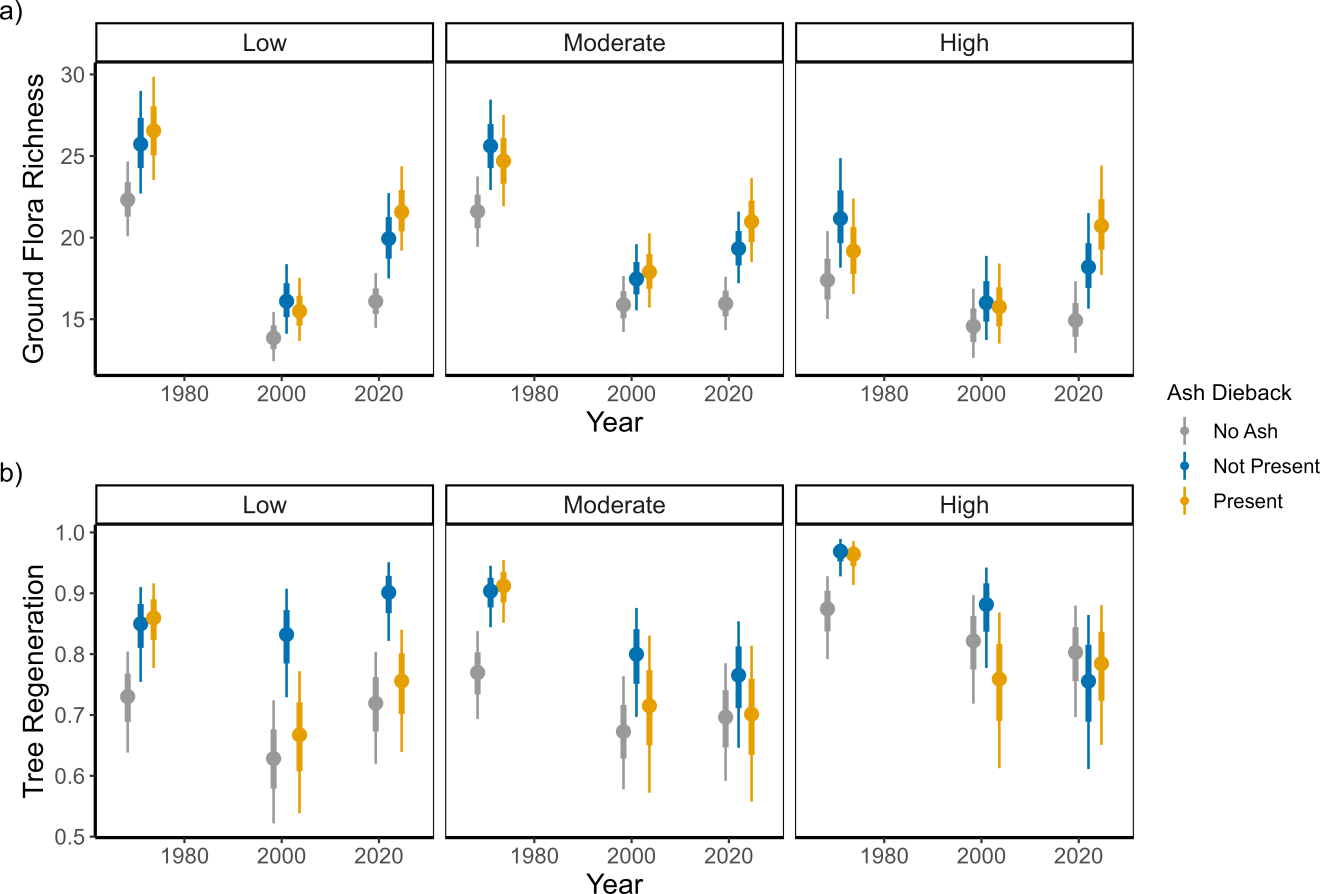
The impact of ash dieback upon species richness in low, moderate and high risk for deer grazing sites. The dot and the thick and thin vertical lines represent the median, 66% interval and 95% interval respectively of the model estimate.

Evaluation of the interaction between ash dieback and deer risk upon ground flora richness showed that there was only a greater increase in ground flora richness under ash dieback compared to ash plots without ash dieback when deer risk was high (figure 4a; electronic supplementary material, table S6). However, in contrast there was higher tree regeneration and richness in plots with no ash dieback and where deer risk was low (figure 4b; electronic supplementary material, table S6). Under higher levels of deer risk the overall levels of tree regeneration and richness was lower and there was no effect of ash dieback. Similarly, ash regeneration increased in locations with no ash dieback present and low deer risk (electronic supplementary materials, figure S3 and table S6).

Ash dieback had no effect upon Shannon diversity under any deer risk level or year, while AWI proportion showed no effect of ash dieback under any level of deer risk for the 2001 and 2022 surveys with a slight tendency for more AWIs under plots that have remained clear of ash dieback and had higher levels of deer risk in 1971 (electronic supplementary materials, figure S4 and table S6). The proportion of plots with ash dieback had no effect upon site-level ground flora richness or proportion of AWI species under any deer risk level, but ground flora richness increased in plots with ash dieback under high deer risk (0.445, 95% interval −0.095 to 1.001; electronic supplementary material, figure S5). Ash dieback increased forb cover under all levels of deer risk but had no effect upon grass or bramble cover under any level of deer risk (electronic supplementary material, table S6).

### (d) Integrative modelling of ground flora change and tree regeneration

**Figure 5.**
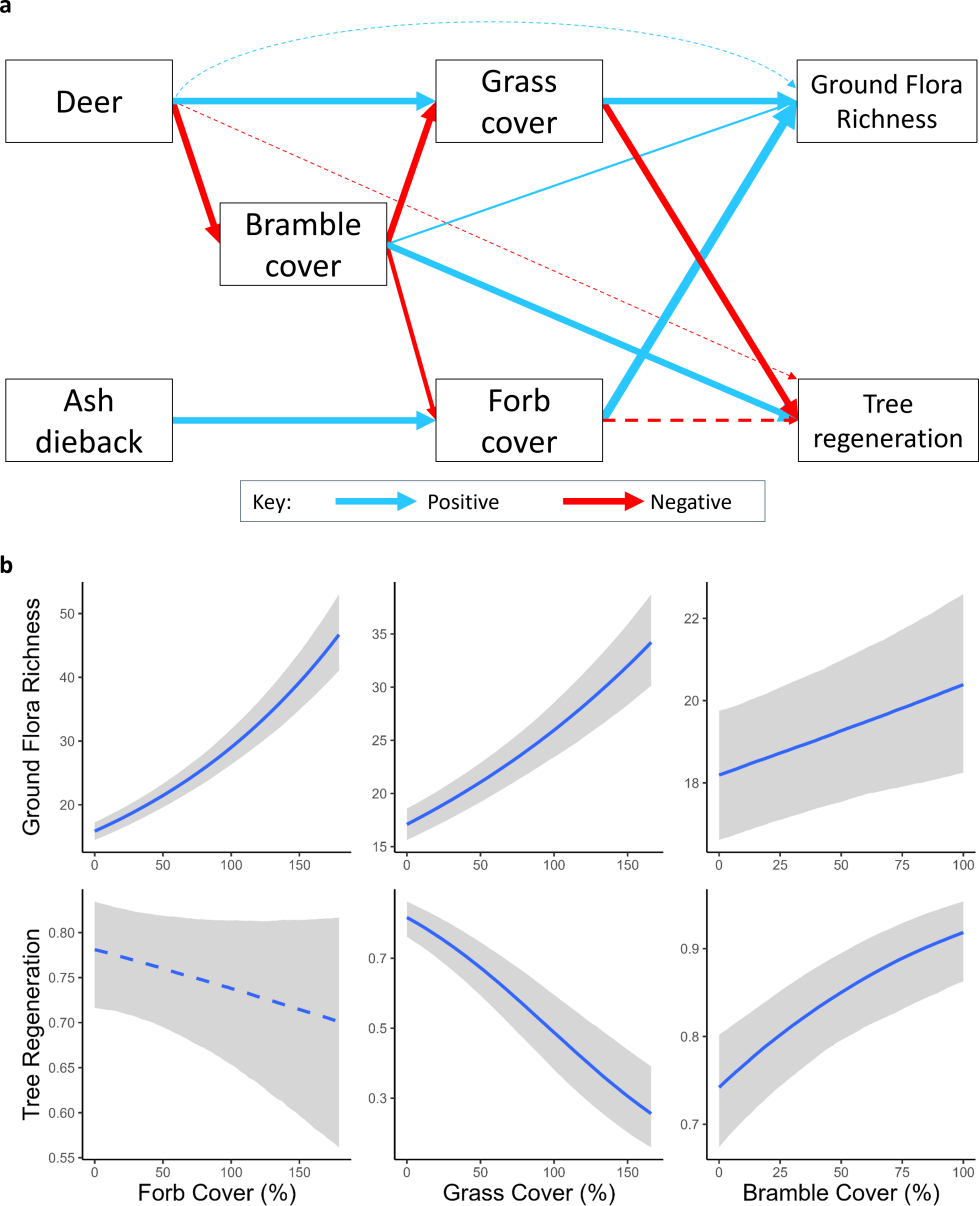
The results of the multivariate model summarized in graphical form (a), and with the effects of forb, grass and bramble cover upon species richness and bramble cover (b). In (a) the boxes represent measured variables, and the arrows modelled relationships with red arrows representing a negative relationship and blue arrows representing a positive relationship. Dashed lines represent relationships where the 95% interval crosses zero, or in the case of the deer effects there being limited additional predictive power from inclusion. The grey-shaded areas in panel (b) show the 95% interval of the effect. The effects of climate and day of year are not shown within this diagram for ease of interpretation, those relationships are shown in electronic supplementary material, figure S6, and all responses of forb, bramble and grass cover shown within this model are shown in electronic supplementary material, figure S7.

Multivariate modelling revealed that the opposing effects of deer risk and ash dieback upon tree regeneration and ground flora richness were largely mediated by changes in bramble, grass and forb cover (figure 5). Ash dieback had no direct effects upon ground flora richness or tree regeneration once changes in climate, day of year, forb, grass and bramble cover were accounted for. There was some indication of direct deer risk effects upon tree regeneration and ground flora richness, as that model performed slightly better than the model with neither a deer risk nor ash dieback effect. However, the difference between the models’ predictive performances was less than the margin of error (electronic supplementary material, table S7). Ground flora richness was positively related to forb and grass cover, with a much weaker positive relationship with bramble cover, while tree regeneration increased strongly with increasing bramble cover and decreased with increasing grass cover (figure 5b). Forb and grass cover both declined with increasing bramble cover (electronic supplementary material, figure S7). The impacts of ash dieback and deer risk upon the cover variables once other variables were accounted for was similar to that within the previously described models, with the exception of the decline in grass cover under low deer risk, which is now entirely explained by the change in bramble cover (electronic supplementary material, figure S7).

Of the original four climate variables (winter rainfall, average winter minimum temperature, summer rainfall and average summer maximum temperature), the model with the best predictive performance included winter rainfall and winter minimum temperature only. Both the model with all climate variables and the model with summer climate only performed similarly to each other, but much more poorly compared to the winter climate only model (electronic supplementary material, table S7). As winter temperature increased, bramble cover and species richness increased, grass cover decreased, while bramble cover and tree regeneration were lower in wetter environments (electronic supplementary material, figure S6). Consistent with the previous results, day of year positively affected bramble cover and grass cover but negatively affected forb cover and species richness (electronic supplementary material, figure S6).

## 4. Discussion

Our results show the importance of considering together the changes in management, disease, herbivory and climate when evaluating the drivers of change in broadleaved woodland ecosystems. The reduction in canopy gap creation from human management, in conjunction with putative widespread canopy recovery from felling during World War II, is visible in both the measured evidence of anthropogenic impacts and the change in tree cohort characteristics. Over the 50 years period stand development has continued towards more shaded and closed canopy forest, in agreement with trends within specific ancient woodlands across Great Britain and Europe [4,6,32,47]. This has led to a much less diverse ground floor community in the twenty-first century compared to the 1970s, as well as lower rates of overall tree regeneration. Shadier conditions are known to lead to non-random loss of species and high rates of woodland homogenization, resulting in a high risk of extinction of many woodland specialist species and a loss of unique woodland ecosystems [6,48,49]. However, the directional change towards shadier conditions has been locally halted by the spread of ash dieback and the associated opening of the canopy. The known site-level effects of disease and grazing are now widespread enough to impact national-level woodland dynamics [50–52]. While the precise impacts of ash dieback upon both the canopy and the ground flora appear to be dependent upon the level of deer grazing, there is in essence a switch from canopy gap creation by humans towards increased canopy gap creation by disease. Since ash is the second most common canopy species in our national sample, these observed effects have the potential to increase further in prevalence and severity.

While we have shown the impacts of ash dieback over the first decade or two after its arrival, there is still potential for the longer-term impacts to differ both quantitatively and qualitatively. Ash trees provide a distinct light, nutrient and hydraulic environment compared to other tree species, with more light being passed through the canopy, higher decomposition rates and greater hydraulic conductivity [21,53]. No tree species has been identified that could replace the functional role of ash in woodland ecosystems [19]. The reduction of ash in the landscape will likely result in changes in woodland communities and ecosystem function due to this distinct functional role of ash and the high numbers of species (particularly invertebrates, fungi and lichens) that are dependent upon it [11,18]. Ash does appear to have some level of tolerance to ash dieback, but the potential invasion of ash borer could result in an even more drastic loss of ash with concomitant effects on European woodlands [14,54]. The loss of ash is also expected to lead to the loss of many invertebrate, fungal and lichen species over the next few decades, which could then feedback to the plant communities [11]. Dieback appears to be worse where ash is more abundant, which could lead to very large openings in the canopy [55]. These openings could be taken over by grass as they outcompete forbs, particularly under high deer pressure, resulting in a further reduction in ash regeneration [53,56,57]. Increases in deer browsing could decrease bramble growth and tree regeneration such that any positive impact on ground flora richness resulting from greater light admitted under infected ash canopies could be accompanied by a lack of canopy renewal as high herbivory removes the youngest stems.

We found that winter climate was more predictive of the ground flora and tree regeneration compared to summer climate. This could be due to winter climate providing a stronger filter on plant survival, or alternatively it could be due to broad-scale winter climate variables being more related to the microclimate experienced by the plants due to the lack of canopy during those months. Microclimate is known to be extremely important in driving vegetation dynamics [23], particularly as recent canopy closure due to changes in forest management has allowed greater buffering of the impacts of macroclimatic warming [24]. The canopy gaps created by ash dieback could, therefore, reduce the ability of forests to cope with climate change. This could also increase sensitivity to tree diseases, as diseases such as ash dieback often have temperature optimums higher than current temperatures [58]. Moreover, climate change will also lead to more extreme weather events creating more gaps in the tree canopy [25,59]. This raises the possibility of a positive feedback process, where gaps are opened in forest canopies based on disease and storm events which then reduce the ability of the forest vegetation to cope with the changing climate and pest invasions. Some plants, such as bramble, may benefit from these changes but others—particularly woodland specialists and shape-adapted species—will lose out [60].

The response of ground flora richness and tree regeneration to ash dieback being dependent upon deer grazing risk as well as influenced by winter climate shows the importance of accounting for multiple drivers, in agreement with experimental studies [60,61]. In British woodlands, several understorey species can exert strong control over species richness [62], of which bramble is one of the most widespread and influential. Our results show that bramble can constrain ground flora response to canopy opening by effectively shading lower-growing species in gaps associated with ash dieback, so long as deer are not present in high enough numbers to keep bramble cover low. At the same time, bramble was also associated with greater tree regeneration. Understanding the response of bramble to other environmental factors such as climate, and identifying the responses of other keystone species, particularly including those animal and fungal species dependent upon ash, is essential when considering how our results will extrapolate to other woodland ecosystems and future novel environmental conditions.

Within this work, we have focused upon the change in summary metrics such as ground flora richness and total tree regeneration, but these could mask changes in composition. Our results show that the increase within plot-level ground flora richness within the twenty-first century is not matched by an increase in richness at the site level, potentially indicating that plant communities within our sites have become more spatially homogenous. Ancient woodlands are known to generally have higher heterogeneity in plant communities than recent forest [63], but it could be that some of this distinctiveness is lost. There is, however, some evidence that site-level ground flora richness is currently greater under high levels of deer grazing and ash dieback. This suggests that deer could be a source of propagules, transporting species between regions and from unsampled areas of the wood, resulting in a wider pool of recorded species within a site that can take advantage of the canopy gap creation [29]. It should be noted that species that may be spread by deer may not be woodland specialists, and this process could lead to a further loss of ancient woodland character as the vegetation composition shifts further from the historic state [64]. We found only limited evidence for changes in the proportion of AWI species at either the plot or site level; however, this could be due to subjectivities in the selection of AWI species and a trait-based approach might yield more informative results [65].

While we have incorporated information on multiple drivers of woodland dynamics into our modelling, including climatic, grazing and disease effects, the complexity and vulnerability of natural ecosystems means that there are always other potential drivers of change. Any causal interpretation of our results relies on a series of assumptions, in particular, we assume that there are no unmeasured confounders and that the directionality of the relationships we have included in our models are correct [66]. The more potentially influential concern is the impacts of unmeasured confounders, which could include the impacts of pollution, human intervention or pre-existing environmental gradients such as soil acidity [3,34,67]. For example, observed increases in species adapted to high fertility environments could be caused by high levels of nitrogen deposition [34]. This could explain some of the increase in ground flora richness in plots with ash, as ash generally occurs in higher fertility environments. Nevertheless, one of the strengths of our analysis, and benefits of long-term monitoring, is that we have information from the sites from before deer populations expanded in the late 1970s and before ash dieback arrived in the early twenty-first century. This means that we can evaluate whether sites with ash dieback were different in species richness from sites with ash but without dieback before ash dieback arrived, which they were not. While it is impossible to fully rule out the possibility of unmeasured environmental effects that happen to coincide with ash dieback and/or deer, the long-term nature of our data enables more confident attribution of responses to changes in ash dieback and deer grazing.

When considering the impacts of ash dieback and the long-term future of Britain’s woodlands, we need to consider the historical context. The opening up of the canopy from ash dieback suggests that the new drivers of woodland dynamics may result in patterns of change that resemble the pulsed gap dynamics resulting from woodland management cycles, where instead of human agents clearing trees we have disease agents doing the same with consequences that may be undesirable from the human perspective. Other novel combinations of global change drivers could still result in novel ecosystem dynamics and dramatic landscape-level change [59,68]. This then raises the problem of how to restore ecosystem function and resilience over the long term under increasing levels of woodland disturbance from disease and other extreme events.

Our analysis confirms that different elements of the woodland ecosystem—ground flora richness versus tree regeneration, for example—respond in different ways to external disturbances, demonstrating the importance of establishing clear goals within woodland management. High forest, with a closed canopy and shaded conditions, will likely have a less diverse plant community, although this trade-off may be acceptable for those focused on maintaining populations of shade-tolerant plants and other biota. In most cases, maintaining a mosaic of lighter and darker conditions is desirable. This is likely to be easier to achieve through natural processes in larger woodlands. In small woods, managers may need to take a more interventionist approach or accept wider fluctuations in species composition and abundance. As efforts continue to stop deforestation and increase afforestation, information on the drivers of long-term dynamics within existing woodlands and the history of change can provide key context for landscape-scale management.

## Data Availability

The data has been made available upon the Environmental Information Data Centre, with 1971–2001 data provided as separate files for ground flora [69], tree data [70] and site information [71], and the most recent survey data provided at [72]. Code is archived at Zenodo [73]. Supplementary material is available online [74].
